# Prediction model for long-term seizure and developmental outcomes among children with infantile epileptic spasms syndrome

**DOI:** 10.3389/fneur.2023.1195252

**Published:** 2023-07-14

**Authors:** Yuto Arai, Tohru Okanishi, Sotaro Kanai, Yuko Nakamura, Hiroshi Sunada, Hinata Murakami, Kazuki Yamauchi, Hisashi Noma, Yoshihiro Maegaki

**Affiliations:** ^1^Division of Child Neurology, Department of Brain and Neurosciences, Faculty of Medicine, Tottori University, Yonago, Japan; ^2^Advanced Medicine, Innovation and Clinical Research Center, Tottori University Hospital, Yonago, Japan; ^3^Faculty of Medicine, Tottori University, Yonago, Japan; ^4^Department of Data Science, The Institute of Statistical Mathematics, Tokyo, Japan

**Keywords:** infantile spasm, developmental delay, prediction model, long-term prognosis, magnetic resonance imaging

## Abstract

**Introduction:**

Children with infantile epileptic spasms syndrome (IESS) are likely to experience poor outcomes. Researchers have investigated the factors related to its long-term prognosis; however, none of them developed a predictive model.

**Objective:**

This study aimed to clarify the factors that influence the long-term prognosis of seizures and their development and to create a prediction model for IESS.

**Materials and methods:**

We conducted a retrospective cohort study enrolling participants diagnosed with IESS at the Tottori University Hospital. We examined the seizure and developmental status at 3 and 7 years after the IESS onset and divided the participants into favorable and poor outcome groups. Subsequently, we analyzed the factors associated with the poor outcome group and developed a prediction model at 3 years by setting cutoff values using the receiver operating characteristic curve.

**Results:**

Data were obtained from 44 patients with IESS (19 female patients and 25 male patients). Three years after epileptic spasms (ES) onset, seizure and development were the poor outcomes in 15 (34.9%) and 27 (61.4%) patients, respectively. The persistence of ES or tonic seizures (TS) after 90 days of onset, moderate or severe magnetic resonance imaging abnormalities, and developmental delay before IESS onset were significantly associated with poor outcomes. Seven years after the onset of ES, seizures and development were the poor outcomes in 9 (45.0%) and 13 (72.2%) patients, respectively. We found that no factor was significantly associated with poor seizure outcomes, and only developmental delay before IESS onset was significantly associated with poor developmental outcomes. Our prediction model demonstrated 86.7% sensitivity and 64.3% specificity for predicting poor seizure outcomes and 88.9% sensitivity and 100% specificity for predicting poor developmental outcomes.

**Conclusion:**

Our prediction model may be useful for predicting the long-term prognosis of seizures and their development after 3 years. Understanding the long-term prognosis during the initial treatment may facilitate the selection of appropriate treatment.

## Introduction

Infantile epileptic spasms syndrome (IESS), which is characterized by epileptic spasms (ES) irrespective of hypsarrhythmia on electroencephalogram (EEG) and neurodevelopmental delay, accounts for 10% of epileptic cases that begin before 36 months of age (1). The incidence of IESS is estimated to be 2–3 per 10,000 live births, and the prevalence is ~0.015 cases per 1,000 people (2, 3). Despite the efficacy of adrenocorticotropic hormone (ACTH), vigabatrin (VGB), and some antiseizure medications, more than half of the patients show severe developmental delay and two-thirds have long-lasting seizures (4, 5).

Seizure control and cognitive status are considered major outcome parameters in any study on IESS (4, 6), and several reports have demonstrated the long-term clinical prognosis of IESS. Regarding seizure prognosis, underlying etiology, persistent ES, and the appearance of epileptic discharges after ACTH therapy are considered the prognostic factors (4, 6). Regarding developmental prognosis, underlying etiology, early treatment initiation, persistent ES, and the presence of focal seizures and developmental delay before the onset of spasms are considered the prognostic factors (4, 6, 7).

Thus, factors associated with prognosis have been identified; however, no study has integrated these factors into a predictive model. Understanding the natural history of childhood-onset epilepsy is imperative for improving the management of the disease and providing children, parents, and families with prognostic information regarding long-term health and social outcomes (8). We aimed to clarify the factors that influence the long-term prognosis of seizures and their development and to create a prediction model.

## Methods

We performed a retrospective cohort study of pediatric patients diagnosed with IESS at the Department of Child Neurology at Tottori University Hospital. These patients were subsequently divided into two groups according to their development and seizure outcomes. Furthermore, we extracted the risk factors associated with poor seizure and developmental prognosis and created predictive models.

### Participants

Data were obtained from the medical records of patients diagnosed with IESS from the Department of Child Neurology at Tottori University Hospital between January 1996 and January 2022. The inclusion criteria were as follows: (i) ES onset at < 36 months of age and (ii) follow-up for >3 years. The exclusion criteria were as follows: (i) patients whose seizures changed to movements that were indistinguishable from TS or hypertonia and (ii) those who developed epileptic encephalopathy in the neonatal period and subsequently developed ES.

We assessed seizures and developmental status at 3 and 7 years after ES onset and divided them into two groups. Regarding seizure outcomes, the poor seizure outcome group was defined as the presence of residual ES or TS, and the favorable seizure outcome group was defined as the absence of seizures for at least 6 months before the date of evaluation or the presence of focal-onset seizures other than ES/TS. Conversely, poor outcomes were defined as intelligence quotient/developmental quotient (IQ/DQ) of < 35, whereas favorable outcomes were defined as IQ/DQ of ≥35.

### Data collection

We collected clinical data, including the sex, age at the onset of ES, the etiology of IESS, seizure and developmental delay before the onset of ES, hypsarrhythmia, family history of epilepsy, and consanguineous marriage within the second degree, days from the onset of ES to the initiation of treatment and disappearance of ES, and the degree of abnormality identified in magnetic resonance imaging (MRI).

The etiologies of IESS were classified into structural, genetic, infectious, metabolic, immune, and unknown types, with reference to the International League Against Epilepsy classification (9). Developmental delay before ES onset was defined as DQ < 70 before the onset of ES (10). The days from ES onset to the beginning of treatment were classified into three categories as follows: < 30 days, 30–89 days, and ≥90 days. The days from ES onset to the resolution were classified into three categories as follows: < 30 days, 30–89 days, and ≥90 days or persistent ES or TS. The degree of MRI abnormality was classified into the following three categories: mild-normal, moderate, and severe (11). The MR images were retrospectively reviewed by two authors (YA and TO).

### Statistical analysis

#### Clinical profiles

We performed Fisher's exact probability tests and Mann–Whitney *U*-tests to compare the clinical profiles among the groups. For these analyses, etiologies were classified into two categories (structural = 0 and non-structural = 1).

#### Prediction model after 3 years of onset

We selected variables with a *p*-value of < 0.05 using Fisher's exact probability tests and Mann–Whitney *U*-tests. Furthermore, we used the receiver operating characteristic (ROC) curve to determine cutoff values for clinical data that demonstrated significant differences between participants with favorable and poor outcomes. We developed a prediction model for the outcomes after 3 years of onset; however, we could not develop a model for the outcomes after 7 years of onset because of fewer participants.

#### Software for statistical analyses and sets of significant and correlation levels

Data analysis was performed using IBM SPSS Statistics version 25.0 (IBM Japan, Tokyo, Japan). A *p*-value of < 0.05 was considered statistically significant.

## Results

### Participants

In total, 48 participants were initially included in the study. Seizures had changed to movements that were indistinguishable from TS or hypertonia in three participants, and one participant developed epileptic encephalopathy in the neonatal period and subsequently developed ES. Finally, we included 44 participants in this study.

Table 1 summarizes the demographics of all participants. The age of ES onset ranged from 3 to 27 months (mean, 8.3 m). Seizures and developmental delay before ES onset were observed in 7 (15.9%) and 24 (54.5%) patients, respectively. Treatment was initiated within 30 days, between 30 and 90 days, and after 90 days of onset in 32 (72.7%), 6 (13.6%), and 6 (13.6%) participants, respectively. We observed structural, genetic, metabolic, infectious, immunological, and unknown etiologies in 17 (38.6%), 5 (11.4%), 4 (9.1%), 2 (4.5%), 0 (0%), and 16 (36.4%) patients, respectively. The degree of MRI abnormalities ranged from normal to mild, moderate, and severe in 20 (45.4%), 6 (13.6%), and 17 (38.6%) participants, respectively.

**Table 1:** Demographic and clinical characteristics of patients after 3 years of ES onset.

**Characteristics**	**Numbers (ratios; total *N* = 44)**
**Gender**	
Male	25 (56.8%)
Onset month age of ES, median (interquartile range)	6 (4–10)
Seizures before ES onset	7 (15.9%)
Developmental delay before ES onset	24 (54.5%)
Hypsarrhythmia	41 (93.2%)
Consanguineous marriage	0 (0%)
Family history of epilepsy	1 (2.3%)
**Days from onset of ES to start of treatment**	
1 (< 30 days)	32 (72.7%)
2 (30–89 days)	6 (13.6%)
3 (≥90 days)	6 (13.6%)
**Days from onset of spasm to resolution**	
1 (< 30 days)	15 (34.1%)
2 (30–89 days)	7 (15.9%)
3 (≥90 days)	22 (50.0%)
**Etiology**	
Structural etiology	17 (38.6%)
CNS malformation	1 (2.3%)
Neurocutaneous disorder	5 (11.4%)
Perinatal brain damage	11 (25.0%)
Genetic etiology	5 (11.4%)
Chromosome abnormalities	5 (11.4%)
Genetic mutation	0 (0%)
Metabolic etiology	4 (9.1%)
Infectious etiology	2 (4.5%)
Congenital CNS infection	1 (2.3%)
Postnatal CNS infection	1 (2.3%)
Autoimmune etiology	0 (0%)
Unknown etiology	16 (36.4%)
**Degree of MRI abnormality** ***N*** = **43**	
1 (mild-normal)	20 (45.5%)
2 (moderate)	6 (13.6%)
3 (severe)	17 (38.6%)

CNS, Central nervous system; ES, epileptic spasms; TS, tonic seizures.

### Statistical analysis

#### Comparison of the outcomes after 3 years of onset

Table 2 summarizes a comparison of the favorable and poor outcome groups after 3 years of onset. The groups with poor seizure and developmental outcomes included 15 (34.9%) and 27 (61.4%) participants, respectively. Regarding seizure and developmental outcomes, we observed significant differences in the sex (*p* = 0.025 vs. *p* = 0.0042), developmental delay before ES onset (*p* = 0.0033 vs. *p* < 0.001), days from ES onset to resolution (*p* = 0.0051 vs. *p* < 0.001), and the degree of MRI abnormalities (*p* = 0.037 vs. *p* = 0.0064).

**Table 2:** Comparison of favorable and poor outcomes groups after 3 years of ES onset.

**Characteristic**	**Seizure outcome (3 years)** ***N*** = **43**			**Developmental outcome (3 years)** ***N*** = **44**		
	**Favorable** ***N*** = **28**	**Poor** ***N*** = **15**	***p*** **-value**	**Favorable** ***N*** = **17**	**Poor** ***N*** = **27**	***p*** **-value**
**Gender**						
Male	16 (57.1%)	7 (46.6%)	0.025^*^	13 (76.5%)	12 (44.4%)	0.0042^*^
Onset month age of ES, median (interquartile range)	6 (4.75–11)	6 (4–8.5)	0.39	6 (4–9.5)	6 (4–9.5)	1
Seizures before ES onset	3 (10.7%)	4 (26.7%)	0.34	1 (5.9%)	6 (22.2%)	0.22
Developmental delay before ES onset	12 (42.9%)	12 (80.0%)	0.0033^*^	1 (5.9%)	24 (88.8%)	< 0.001^*^
Hypsarrhythmia	28 (100%)	13 (86.7%)	0.33	17 (100%)	24 (88.8%)	0.27
Consanguineous marriage	0 (0%)	0 (0%)	NA	0 (0%)	0 (0%)	NA
Family history of epilepsy	0 (0%)	1 (6.7%)	0.35	0 (0%)	1 (3.7%)	1
**Days from ES onset to start of treatment**			0.59			0.83
1 (< 30 days)	20 (71.4%)	12 (80%)		12 (70.6%)	20 (74.1%)	
2 (30–89 days)	4 (14.3%)	2 (13.3%)		4 (23.5%)	2 (7.4%)	
3 (≥90 days)	4 (14.3%)	1 (6.7%)		1 (5.9%)	5 (18.5%)	
**Days from ES onset to resolution**			0.0051^*^			< 0.001^*^
1 (< 30 days)	13 (46.4%)	2 (13.3%)		10 (58.8%)	5 (18.5%)	
2 (30–89 days)	6 (21.4%)	1 (6.7%)		4 (23.5%)	3 (11.1%)	
3 (≥90 days/persistent ES/TS)	9 (32.1%)	12 (80.0%)		3 (17.6%)	19 (70.3%)	
**Etiology**			0.51			0.124
Structural	9 (32.1%)	7 (46.7%)		4 (23.5%)	13 (48.1%)	
Non-structural	19 (67.9%)	8 (53.3%)		13 (76.5%)	14 (51.9%)	
**Degree of MRI abnormality** ***N*** = **43**			0.037^*^			0.0064^*^
1 (mild-normal)	17 (60.7%)	3 (20.0%)		13 (76.5%)	7 (25.9%)	
2 (moderate)	2 (7.1%)	4 (26.7%)		0 (0%)	6 (22.2%)	
3 (severe)	9 (32.1%)	8 (53.3%)		4 (14.9%)	14 (51.8%)	

ES, epileptic spasms; TS, tonic seizures; NA, not available.

^*^p < 0.05.

#### Comparison of the outcomes after 7 years of onset

Table 3 summarizes a comparison of the favorable and poor outcomes groups after 7 years of onset. We followed up on seizures and developmental status in 20 and 18 participants, respectively, up to 7 years after onset. In total, 9 (45.0%) and 13 (72.2%) participants demonstrated poor seizure and developmental outcomes, respectively. We found that no symptoms were significantly associated with poor seizure outcomes, and only developmental delay before IESS onset was significantly associated with poor developmental outcomes (*p* = 0.0025). All participants with poor seizure and developmental outcomes at 7 years also demonstrated poor seizure and developmental outcomes at 3 years.

**Table 3:** Comparison of favorable and poor outcomes groups after 7 years of ES onset.

**Characteristic**	**Seizure outcome (7 years)** ***N*** = **20**			**Developmental outcome (7 years)** ***N*** = **18**		
	**Favorable** ***N*** = **11**	**Poor** ***N*** = **9**	***p*** **-value**	**Favorable** ***N*** = **5**	**Poor** ***N*** = **13**	***p*** **-value**
**Gender**						
Male	8 (72.7%)	4 (44.4%)	0.36	4 (80.0%)	7 (53.8%)	0.6
Onset month age of ES, median (interquartile range)	5 (5–9.5)	6 (4–9)	0.67	7 (5–10)	5 (4–9)	0.3
Seizures before ES onset	2 (18.2%)	3 (33.3%)	0.62	1 (20.0%)	4 (30.8%)	1
Developmental delay before ES onset	4 (36.4%)	7 (77.8%)	0.092	0 (0%)	11 (84.6%)	0.0025^*^
Hypsarrhythmia	11 (100%)	8 (88.9%)	0.45	5 (100%)	12 (92.3%)	1
Consanguineous marriage	0 (0%)	0 (0%)	NA	0 (0%)	0 (0%)	NA
Family history of epilepsy	0 (0%)	1 (11.1%)	0.45	0 (0%)	1 (7.7%)	1
**Days from ES onset to start of treatment**			0.16			0.17
1 (< 30 days)	4 (36.4%)	6 (66.7%)		2 (40.0%)	8 (61.5%)	
2 (30–89 days)	3 (27.3%)	2 (22.2%)		2 (40.0%)	2 (15.4%)	
3 (≥90 days)	4 (36.4%)	1 (11.1%)		1 (20.0%)	3 (23.1%)	
**Days from ES onset to resolution**			1			0.42
1 (< 30 days)	0 (0%)	2 (22.2%)		0 (0%)	2 (15.4%)	
2 (30–89 days)	3 (27.3%)	0 (0%)		3 (60.0%)	0 (0%)	
3 (≥90 days/persistent ES/TS)	8 (72.7%)	7 (77.8%)		2 (40.0%)	11 (84.6%)	
**Etiology**			1			0.6
Structural	4 (36.4%)	3 (33.3%)		1 (20.0%)	6 (46.2%)	
Non-structural	7 (63.6%)	6 (66.7%)		4 (80.0%)	7 (53.8%)	
**Degree of MRI abnormality** ***N*** = **43**			0.34			0.153
1 (mild-normal)	7 (63.3%)	3 (33.3%)		4 (80.0%)	4 (30.8%)	
2 (moderate)	1 (9.1%)	3 (33.3%)		0 (0%)	4 (30.8%)	
3 (severe)	3 (27.3%)	3 (33.3%)		1 (20.0%)	5 (38.5%)	

ES, epileptic spasms; TS, tonic seizures; NA, not available.

^*^p < 0.05.

#### Prediction of seizure and developmental outcomes after 3 years of onset

**Figure 1:**
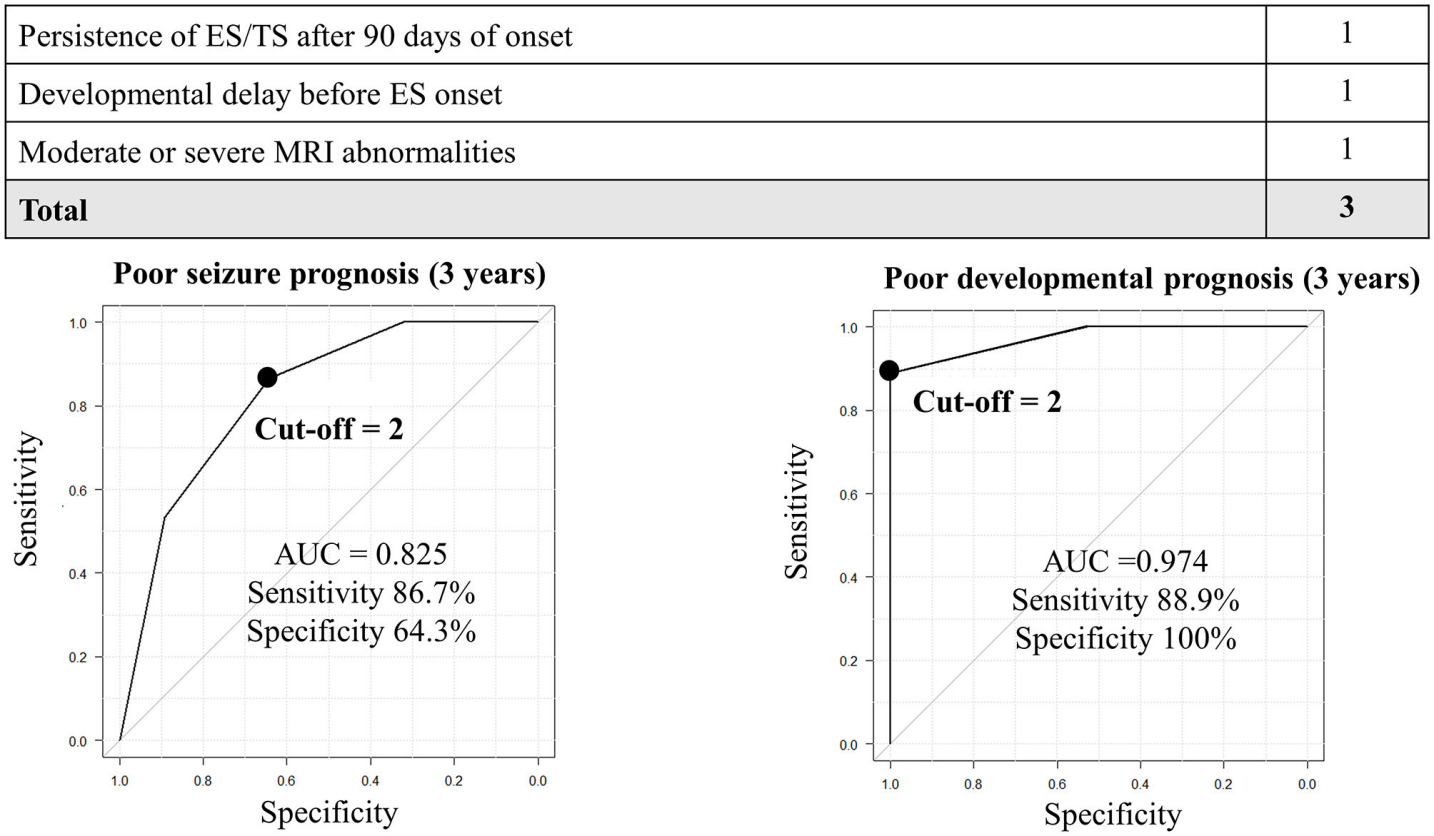
Prediction of seizure and developmental prognosis after 3 years of ES onset using clinical data. In the prediction model for poor seizure prognosis, the cutoff point of 2 generates a sensitivity and specificity of 60 and 89.3%, respectively. In the prediction model for poor developmental prognosis, the cutoff point ≥1 generates a sensitivity and specificity of 100 and 76.5%, respectively. AUC, area under the receiver operating characteristic curve.

Before establishing a prediction model for poor seizure outcomes, we set cutoff values of the days from ES onset to the resolution and degree of MRI abnormalities using ROC curves, which generated a score of 3 [persisting ES/TS after 90 days of onset; sensitivity: 80.0%, specificity: 67.9%, and area under ROC (AUC): 0.74] and 2 (moderate; sensitivity: 80.0%, specificity: 60.7%, and AUC: 0.68), respectively. Subsequently, we plotted a ROC curve integrating one point for “persisting ES/TS after 90 days of onset,” “developmental delay before ES onset,” and “moderate or severe MRI abnormalities.” A cutoff value of 2 points demonstrated a sensitivity of 86.7% and a specificity of 64.3% (AUC: 0.825; Figure 1).

Similarly, before establishing a prediction model for poor developmental outcomes, we set cutoff values of the days from ES onset to the resolution and the degree of MRI abnormalities using ROC curves, which generated a score of 3 (persisting ES/TS after 90 days of onset; sensitivity: 70.4%, specificity: 82.4%, and AUC: 0.76) and 2 (moderate; sensitivity: 74.1%, specificity: 76.5%, and AUC: 0.73). The ROC curve was created by integrating with one point for “persisting ES/TS after 90 days of onset,” “developmental delay before ES onset,” and “moderate or severe MRI abnormalities.” A cutoff value of 2 points demonstrated a sensitivity of 88.9% and a specificity of 100% (AUC: 0.974; Figure 1).

In total, 13 participants presented with moderate or severe MRI abnormalities with persisting ES/TS after 90 days of onset, and nine (69%) demonstrated poor seizure outcomes (Supplementary Table 1). Notably, 16 participants presented with developmental delay before ES onset; ES/TS persisted for >90 days, and all (100%) demonstrated poor developmental outcomes (Supplementary Table 2). In contrast, 13 participants did not demonstrate developmental delay before ES onset; ES ceased within 90 days, and all (100%) demonstrated favorable developmental outcomes (Supplementary Table 3).

## Discussion

We clarified the factors that influence the long-term outcomes of seizures and development and developed a prediction model after 3 years of the ES onset. We could not develop the prediction model after 7 years of ES onset; however, all participants with poor seizures and development at 7 years also demonstrated poor seizures and development at 3 years.

Persisting ES/TS after 90 days of onset was an important predictor of both seizure and developmental outcomes. Prolonged ES/TS indicates extensive damage from the cerebrum to the brainstem due to the underlying pathological mechanisms of ES involving both the cortex and subcortical structures, including the brainstem, thalamus, and basal ganglia (12, 13). Therefore, if seizures are refractory to the initial ACTH therapy, clinicians should promptly initiate the subsequent treatments for IESS, including second ACTH therapy, VGB, surgical interventions, and dietary therapy (14–17).

Moreover, moderate or severe MRI abnormalities are important predictors of both seizure and developmental prognoses. MRI may provide complementary information regarding etiology and seizure and developmental prognosis in IESS, particularly when assessed together with EEG (11). Indeed, 69.2% of the participants with moderate or severe MRI abnormalities demonstrated poor seizure outcomes, due to underlying etiologies (Supplementary Table 1). However, the outcome of participant 4 with TSC may have been improved by early VGB initiation (Supplementary Table 1). Corpus callosotomy and focal resection for IESS with unilateral or bilateral lesions have become widespread (15–19). Therefore, clinicians should consider the most appropriate treatment including for moderate or severe degrees of MRI abnormality.

Developmental delay before ES onset is an important predictor of both seizures and developmental prognosis. Classically, if patients with IESS have an identified etiology and/or substantial developmental delay before ES onset, they are categorized into the symptomatic infantile spasms group, which generally demonstrates poor developmental outcomes (4, 20). However, some patients with IESS and precedent developmental delay have structural and metabolic etiologies that can improve developmental prognosis with early intervention (20, 21). We should carefully consider the development potential and not easily determine ES treatment including in patients with precedent developmental delay.

In contrast, all patients in our study demonstrated favorable developmental outcomes if there was no developmental delay before ES onset and ES disappeared within 90 days (Supplementary Table 3). Therefore, early ES cessation is crucial, particularly in patients without developmental delay before ES onset, and clinicians are required to develop a treatment plan in cases with no responses to the initial treatment (4).

This study has several limitations. First, this study included only 44 participants from a single site because this was an exploratory study. Second, we did not evaluate the generalizability of the model on new data. Third, variables were selected by univariate screening. Therefore, larger prognostic research and validation of the generalizability of the model should be considered in future research.

## Conclusion

Our scoring system may be useful for predicting the long-term prognosis after 3 and 7 years of ES onset. Understanding the long-term prognosis of seizures and their development during the initial treatment may facilitate appropriate treatment selection. Moreover, the early cessation of ES leads to improved long-term prognosis for both seizures and development, thus emphasizing research exploring effective treatments for ES.

## Data availability statement

The raw data supporting the conclusions of this article will be made available by the authors, without undue reservation.

## Ethics statement

The studies involving human participants were reviewed and approved by Institutional Ethics Committee of the Tottori University Hospital (approval number: 22A108). Written informed consent from the participants' legal guardian/next of kin was not required to participate in this study in accordance with the national legislation and the institutional requirements.

## Author contributions

TO was responsible for the organization and coordination of the trial. YA was the chief investigator responsible for data analysis. HN was responsible for data analysis. SK, YN, HS, HM, KY, and YM designed the trial. All authors have contributed to the writing of the final manuscript and met the ICMJE authorship criteria.
